# High-Pressure Processing of Fruit Smoothies Enriched with Dietary Fiber from Carrot Discards: Effects on the Contents and Bioaccessibilities of Carotenoids and Vitamin E †

**DOI:** 10.3390/molecules29061259

**Published:** 2024-03-12

**Authors:** Melisa Donda Zbinden, Mario Schmidt, Charito Ivana Vignatti, María Élida Pirovani, Volker Böhm

**Affiliations:** 1 Instituto de Tecnología de Alimentos, Universidad Nacional del Litoral, Santa Fe 3000, Argentina; mdondazbinden@fiq.unl.edu.ar (M.D.Z.); cvignatti@fiq.unl.edu.ar (C.I.V.); mpirovan@fiq.unl.edu.ar (M.É.P.); 2 Consejo Nacional de Investigaciones Científicas y Técnicas (CONICET), Santa Fe 3000, Argentina; 3 Institute of Nutritional Sciences, Friedrich Schiller University Jena, 07743 Jena, Germany; mario.schmidt@uni-jena.de

**Keywords:** by-product, functional foods, beverages, secondary plant products, in vitro bioaccessibility

## Abstract

The effects of high-pressure processing (HPP) (450 MPa/600 MPa/3 min) on the carotenoid and vitamin E contents of smoothies made from strawberry, orange juice, banana and apple, and the same smoothies enriched with dietary fiber from discarded carrots were compared. The contents and bioaccessibilities of these compounds were also evaluated over the course of 28 days at 4 °C. The application of HPP in the formulations significantly increased the contents of β-cryptoxanthin, α-carotene and β-carotene and retained the contents of lutein, zeaxanthin and vitamin E compared to untreated samples. A decreasing trend in the content of each compound was observed with an increase in storage time. The application of HPP initially led to reductions in the bioaccessibility of individual compounds. However, overall, during storage, there was an increase in bioaccessibility. This suggests that HPP influences cell structure, favoring compound release and micelle formation. HPP is a sustainable method that preserves or enhances carotenoid extractability in ready-to-drink fruit beverages. Furthermore, the incorporation of dietary fiber from carrot processing discards supports circular economy practices and enhances the health potential of the product.

## 1. Introduction

Foods that we consume daily provide not only essential macronutrients necessary for life, but also other non-nutritive compounds for health promotion and disease prevention [1]. These non-nutritive bioactive plant compounds in fruits, vegetables, grains and other plant-based foods are known as phytochemicals and give rise to a very promising developing area of research, forming part of numerous research studies for applications in food industry, modern pharmacology, agrochemistry, cosmetics and nano-bioscience. Bioactive compounds (BCs) can be defined as non-nutritive substances of food origin that have a biological activity and can interact with one or more components of living tissue to achieve favorable effects on the health of the organism, depending on the substance, the dose or its bioavailability [2].

Among the BCs provided by plant-based foods are hydrophilic compounds (phenolic compounds and vitamin C) and lipophilic compounds (carotenoid compounds and vitamin E) [3]. Carotenoid compounds are potent antioxidants that can be classified according to their chemical structure into carotenes and xanthophylls. They are lipophilic organic pigments of the isoprenoid group, responsible for the color of many vegetables [4]. Vitamin E compounds are recognized antioxidants, supplied in high concentrations by some plant foods, and have been linked to a lower risk of cancer and cardiovascular diseases [5,6,7,8]. In recent years, increased consumer awareness of the benefits of consuming fruit and vegetables has resulted in changes in dietary behavior. Furthermore, a diet enriched in these compounds may offer a greater and more diverse group of BCs than those available via conventional supplementation [3]. Some authors have associated the biological activity of these compounds with a reduced risk of chronic diseases such as cancer and inflammatory diseases [9,10,11,12].

The demand for fresh, healthy and easy-to-eat foods has generated in the food industry the need to apply new preservation techniques that can extend the shelf life of the products and, at the same time, increase their nutritional and bioactive potential, while maintaining its sensory attributes [13,14,15,16]. Considering that fruits and vegetables can be used as a whole as well as in pieces, or as raw material to prepare other products, the industry has been trying for some time to develop new presentations. If the focus is on consumers who are particularly attracted to liquid or semi-liquid, ready-to-drink products, smoothies are promising candidates for the food industry. A smoothie results from the blending of fruits and vegetables which, after undergoing unit operations, are transformed into a beverage with a typically smooth, semi-liquid consistency. Smoothies can be considered examples of so-called “superfoods” because they represent a convenient way to quickly ingest health-promoting BCs in a fast-paced daily life [17,18]. Strawberries and oranges are fruits popularly demanded by consumers and highly appreciated for their taste, attractive color and health benefits, and can be used to prepare smoothies. The antioxidant, anticarcinogenic and anti-inflammatory characteristics of these fruits are associated with their phenolic and carotenoid compounds and their high vitamin C content [19,20]. In this study, in order to increase the nutritional profile of smoothies, a by-product of the food industry using a fiber powder from carrot discard was added [21]. In previous works, it was demonstrated that this by-product provided a good sensory acceptability by consumers when it was used daily [22,23], and it improved glucose control and reduced body weight and plasma lipid concentrations in normal rats [24].

Conventional heat treatment is the preservation method generally used to ensure the safety of juices and beverages [25]. A disadvantage of conventional heat treatment is the changes in sensory and nutritional attributes that occur in these products after application [26]. Therefore, new processing technologies are required to ensure the preservation of sensory, nutritional and bioactive characteristics of foods without compromising food safety [27]. High-pressure processing (HPP) satisfactorily meets these requirements. Moreover, it is an emerging technique that shows reduced environmental impacts in terms of energy demand and CO_2_ emissions relative to conventional pasteurization and in terms of water requirements [15,28,29]. In addition, HPP improves the extractability of bioactive compounds from the vegetable matrix, promoting a circular economy in the food processing industry [30,31] and meeting the Sustainable Development Goals defined by the United Nations.

Gentle treatment via HPP consists of applying pressures between 100 and 900 MPa for short times to a liquid, usually water, containing the packaged solid and liquid foods. HPP reduces or eliminates pathogenic and spoilage microorganisms, as well as the activity of certain enzymes, without the use of preservatives and/or chemical additives, at a lower temperature compared to conventional heat treatment and without causing loss of food quality (flavor, aroma, color, nutrients, bioactive compounds). Consequently, fruit and vegetable juices treated by means of HPP can be perceived as more natural and healthier [32,33,34,35]. The effect of the application of a high pressure in fruit-based smoothies has been studied [32,33,36]. However, as the effects of HPP vary depending on the selection of fruits and/or vegetables used in the formulation conditioning their pH, soluble solids, enzymatic activity, and microbiological quality during storage, it is necessary to carry out specific studies on each type of product [34]. At the same time, it is interesting to evaluate the possibility of using a milder HPP treatment (450 MPa) than the commercially available one (600 MPa) to potentially reduce industrial costs while ensuring the microbiological and health-promoting quality of the product.

While it is important to find technologies to ensure the safety of a food and to preserve its health-promoting potential, the biological activity of a BC is not only determined by its amount inside a product, but also by its bioaccessibility. The bioaccessibility of a BC is the fraction that is released from the food matrix during gastrointestinal digestion and is available for intestinal absorption [37]. The bioaccessibility of carotenoids and vitamin E occurs when these lipophilic compounds are transferred from the food matrix to mixed micelles during digestion, as through this process the compounds become accessible for apical absorption by the intestinal mucosa. Prior to absorption in the small intestine, these compounds must first be released from the food matrix and then solubilized in small oil droplets and incorporated into micelles [38]. In general, the bioaccessibility of carotenoid compounds is relatively low with respect to the content at which the compound is found in the undigested matrix; in addition, the bioaccessibility of individual carotenoids is affected by various factors such as solubility (facility to transfer to mixed micelles), the processing to which the food is subjected for preservation, interactions with other macromolecules or between carotenoids, and the presence of dietary fat [38,39].

The high content of polyphenols, vitamin C and antioxidant capacity in smoothies of the same or similar fruit composition [40] has been previously described. However, to the best of our knowledge, only a few studies have addressed the profile of lipophilic bioactive compounds in matrices with a high water content [32,33,36]. In this way, the aim of this study was to determine the carotenoid and vitamin E contents of a food matrix composed of several popular fruits, and how industrial processing influences the preservation of this type of product. Hence, a solvent extraction method [41] was used to identify and quantify carotenoid compounds and vitamin E using HPLC-DAD/FLD. The bioaccessibility after processing and during refrigerated storage was also studied using an in vitro digestion assay adapted to this type of food matrix [37,42,43]. In addition, the effects of the non-thermal pasteurization treatment and the incorporation of an innovative food additive, a dietary fiber powder derived from discarded carrots, on the content and bioaccessibility of BCs were evaluated in fruit smoothies.

## 2. Results and Discussion

### 2.1. Identified Carotenoids

**Figure 1:**
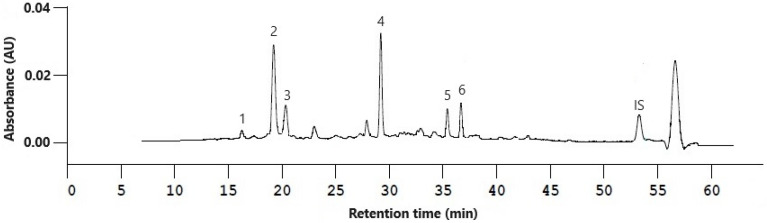
Chromatogram of smoothie extract prior to high-pressure processing. Identified compounds are listed in Table 1. Lycopene was used as internal standard (IS, t_R_ = 53.44 min). A gradient peak caused by LC equilibration appears after 56.69 min.

The identification of carotenoids in smoothie extracts according to their retention time, absorption wavelengths and mass-to-charge ratio via RP-HPLC-DAD/MS measurements is presented in Table 1. The obtained data were compared to literature references on carotenoid identification and to the analysis of extracts from each of the individual fruits constituting the smoothie formulation [44,45,46,47,48]. A chromatogram of a smoothie extract before HPP treatment at a wavelength of 450 nm with lycopene as the internal standard (IS) is shown in Figure 1. Four xanthophylls (peaks 1–4), and two carotenes (peaks 5 and 6) were identified (Figure 1). The carotenoid extraction procedure was also performed on carrot fiber powder to verify the contribution of individual carotenoids to the analysis by this additive. None of the investigated compounds were present in carrot fiber powder.

The xanthophylls identified in smoothies were (*all-E*)-antheraxanthin, (*all-E*)-lutein, (*all-E*)-zeaxanthin and (*all-E*)-β-cryptoxanthin and the carotenes identified were (*all-E*)-α-carotene and (*all-E*)-β-carotene. (*all-E*)-Antheraxanthin could not be quantified in the analyses because it was always below the detection limit, so it will not be considered in the discussion of the results. Table 2 shows the initial carotenoid contents of the smoothies (BF and FF) before treatment via HPP. β-Cryptoxanthin and lutein were the main carotenoids in the untreated smoothies, followed by β-carotene and zeaxanthin and lastly α-carotene.

### 2.2. Effects of Processing and Storage on Carotenoids

The carotenoids most frequently found in fruits and vegetables are β-carotene, α-carotene, β-cryptoxanthin, lycopene, lutein and zeaxanthin [49]. The exhaustive study of the behavior of these compounds regarding food processing is a challenge for researchers to increase the health potential of products for daily consumption. In this way, the effect of processing on the individual carotenoid concentration found in the smoothie’s formulation is shown in Table 2.

Lutein, whose presence is mostly caused by including orange and strawberry, was retained after HPP treatment compared to untreated samples. Lutein resistance to HPP treatments has been reported in apricot nectar subjected to 400 MPa and 500 MPa at 25 °C for 5, 10, and 15 min [50] and in carrot juice after the application of 550 MPa at 25 °C for 6 min [51], while it has also been reported in green beans and broccoli subjected to 400 MPa and 600 MPa at 25 °C for 2 min, and no effect (*p* > 0.05) of HPP application on lutein content was recorded [52]. Additionally, in rosehip puree, no effect of the application of 200, 400, and 600 MPa for 5 or 10 min at room temperature (*p* > 0.05) on lutein content was observed [53].

The presence of zeaxanthin in our smoothies is attributed to the orange juice content present in them, and it was maintained after HPP treatment in both formulations, showing no significant differences (*p* > 0.05) compared to the untreated samples. Similar behavior with regard to zeaxanthin was noted in orange juice subjected to 350 MPa at 30 °C for 15 min, where no significant differences (*p* > 0.05) were found after processing in comparison to untreated juice [54] as well as in orange juice (Navel and Cara Cara orange juice) subjected to 400 MPa at 25 °C for 1 min [55]. Zeaxanthin retention was also found in apricot nectar after application of 400 MPa at 25 °C for 10, 15 and 20 min and at 500 MPa at 25 °C for 5, 10, 15 and 20 min [50]. In rosehip puree, no effect of the application of 200, 400 and 600 MPa for 5 or 10 min at room temperature (*p* > 0.05) on zeaxanthin content was observed [53]. Westphal et al. [53] suggested that the stability of lutein and zeaxanthin after HPP may be attributed to the fact that these compounds are more strongly bound to the plant matrix than the other individual compounds in the food matrix. 

Regarding the pro-vitamin A carotenoids—specifically, β-carotene, α-carotene, and β-cryptoxanthin, which can be enzymatically cleaved to produce vitamin A—the effect of HPP application on the extractability of these compounds was observed (*p* ≤ 0.05). Under both pressure conditions as well in BF and FF formulations, an increase in the individual contents of these carotenoids was detected. The presence of β-cryptoxanthin in our formulations comes from orange juice. In the case of β-cryptoxanthin, an increase in concentration of this compound was observed (*p* ≤ 0.05) after the application of 450 MPa and 600 MPa. Interestingly, some authors also found increases after HPP that were significant. De Ancos et al. [54] showed that this increase could also be quantified in orange juice subjected to 350 MPa for 5 min at 30 °C, determining an increase of 42%. Jacobo-Velázquez and Hernández-Brenes [56] also found a 220% increase in β-cryptoxanthin content in avocado paste after the application of 600 MPa for 3 min at 23 °C. 

The presence of α-carotene in our smoothies is mainly due to orange juice, followed by lower quantities found in the banana used in the formulations. An increase of approximately 40–48% was observed for BF and FF, respectively, after the application of 450 MPa, and 600 MPa. There is no significant difference (*p* > 0.05) between both formulations and both pressure conditions. Jacobo-Velázquez and Hernández-Brenes [56] reported much higher increases (almost 400%) after the application of 600 MPa for 3 min at 23 °C in avocado paste. In orange juice subjected to 350 MPa for 5 min at 30 °C, an increase (*p* ≤ 0.05) of 60% in this carotenoid compound was determined [54]. In apricot nectar, an increase of approximately 100% in this compound (*p* ≤ 0.05) was found after the application of 300 MPa for 5, 10, 15, or 20 min and after the application of 400 MPa for 5 min at 25 °C [50]. After the application of HPP to pumpkin cubes for 3 min using cold water (3–4 °C), an increase (*p* ≤ 0.05) of 100% using 400 MPa and 168% using 600 MPa was determined [57]. In the present study, we found lower percentage increases than those cited above. This could be because α-carotene has different degrees of extractability depending on the plant matrix on which the high pressures are applied. The extractability of α-carotene is higher after HPP on individual matrices compared to the application of HPP on mixed matrices formed by the addition of different fruits or vegetables. In smoothies containing orange juice, papaya juice, melon juice, carrot puree and skim milk, no significant increase (*p* > 0.05) in the content of α-carotene was observed after the application of 450 and 600 MPa for 3 min at 20 °C [33]. In our case, α-carotene was retained after processing.

The presence of β-carotene in these formulations is mainly due to orange juice. An increase in β-carotene of 35% and 10% was observed for BF and FF, respectively, after the application of 450 MPa, and an increase in β-carotene of 29% and 21% for BF and FF, respectively, after the application of 600 MPa. Andres et al. [33] reported results that agree with ours, and their smoothies showed an increase in β-carotene extractability (*p* ≤ 0.05) of 13% after the application of 450 MPa and 25% after the application of 600 MPa at 20 °C. Then, a significant increase (*p* ≤ 0.05) was also found after application of 600 MPa for 10 min to melon pieces [54] and an increase (*p* ≤ 0.05) of 73% after the application of 400 MPa and 95% after the application of 600 MPa to pumpkin cubes for 3 min at 20 °C [58]. Our results are consistent with Ancos et al. [54], who found a 50% increase in β-carotene in orange juice subjected to 350 MPa for 5 min at 30 °C. 

Changes in extractability are most probably the main cause of an increase in bioactive compounds contents. The alteration in molecular volume caused by the application of pressure (>150 MPa), governed by Le Chatelier’s principle, has a strong impact on the structure of the plant cell membrane, enhancing the extractability of intracellular components [59]. HPP induces changes in cell membrane permeability that result in a response like that generated by mechanical stress-induced damage in plant tissues. The application of HPP causes damage to cell membranes, resulting in the release of ATP from the cell cytoplasm. Then, the binding of ATP to undamaged cells generates immediate, early or late responses [15,59]. On the other hand, the retention of or increase in extractability of individual carotenoids can be attributed to the degree of association of each individual carotenoid with the macromolecule to which it is bound, forming stronger or weaker bonds. At pressures higher than 300 MPa, denaturation of the proteins present in the food matrix occurs at room temperature [54], so higher pressures would allow for obtaining higher percentages of extractability for certain compounds, as could be observed in this study with β-cryptoxanthin, α-carotene and β-carotene. In agreement with this, following the study of HPP application on rosehip puree, Westphal et al. [53] suggested that the extraction capacity of each individual carotenoid is conditioned by the way in which each carotenoid is bound in protein–carotenoid complexes and associated with the plant matrix and by the processing of the plant matrix before HPP application (cutting, milling, heat treatment, etc.).

Table 2 also shows the change in the contents of individual carotenoids in the HPP samples during storage at a refrigerated temperature of 4 °C. For all compounds, a consistent decrease was observed, reflecting the impact of storage. Throughout the storage period, there was a degradation trend in the compounds, with accentuation noted in the final storage period until day 28. This trend aligns with findings in carrot juice subjected to 550 MPa for 6 min and stored for 20 days at 4 °C, where a decrease in carotenoids was observed with an increase in storage time. This phenomenon was attributed to light incidence and dissolved oxygen concentration inside the HHP-treated containers [51]. Notably, in our case, light incidence was not a contributing factor, as the samples were stored under dark conditions.

After 14 days, in FF treated at 450 MPa and at 600 MPa and in BF treated at 600 MPa, lutein was retained (*p* > 0.05) when compared to the sample at day 0. However, a decrease in lutein concentration was observed until the end of the storage period on day 28. This behavior was also seen for zeaxanthin and α-carotene. In agreement to smoothies preserved at 4 °C and subjected to comparable pressure conditions and treatment times to those in the present study, similar decreasing trends were observed for α-carotene and β-carotene until the 45th storage day [33], attributed to storage causing inactivation of enzymes that result in the loss of carotenoids.

During the storage of smoothies, oxygen exists mainly in the form of triplet oxygen (^3^O_2_), which can mainly be divided into oxygen in the headspace of the package, dissolved oxygen in the plant matrix and diffuse oxygen entering the package through the packaging materials. Dissolved oxygen can easily cause the oxidation of lipophilic compounds [3]. While in storage, fruit and vegetable products are inevitably exposed to natural light or artificial illumination which can lead to a decrease in shelf-life quality. The effect of light incidence on the product depends not only on the characteristics of the vegetable matrix but also on the wavelength, light intensity and light-blocking properties of the packaging materials [3]. Another possible cause of carotenoid degradation during storage may be due to structural changes such as isomerization caused by the application of HPP [49]. During the storage of tomato pulp at 5 °C, a decrease of 35% in lycopene content was determined when storage time was longer than 15 days and was attributed to isomerization and oxidation reactions [60]. Organic acids, heat and light promote isomerization of the most common configuration found in nature: (*all-E*)-carotenoids. The cutting, pulping and squeezing of fruits and, to a greater extent, food processing result in E-Z isomerization. An example of degradation of an (*all-E*)-carotenoid by processing that favors isomerization of the compound occurs after the application of ultrasound processing. Song et al. [61] showed that the application of ultrasonic waves causes the isomerization of (*all-E*)-lutein to its isomers (*13-Z*)-lutein, (*13′-Z*)-lutein, (*9-Z*)-lutein and (*9′-Z*)-lutein. Vervoort et al. [62] showed that sterilization by means of heat and high-pressure treatments generate *Z-*isomers of β-carotene.

On the other hand, β-cryptoxanthin showed a significant increase in concentration at 14 days of storage. In avocado paste subjected to 600 MPa for 3 min, an increase in this compound was observed after 10 days of storage at 4 °C. The processing probably affected the structure of the carotenoid-containing chloroplasts and the permeability of the cell membrane resulting in a release of the carotenoid during subsequent storage of the product [56].

### 2.3. Effects of Processing and Storage on Vitamin E

Only a few studies have investigated the α-tocopherol content in fruit-based products such as fruit smoothies. Less research has been conducted on the influence of HPP on α-tocopherol in fruit juices or smoothies. It is therefore interesting to expand the field of research in this direction to contribute to the food processing industry and to establish the health potential of everyday consumer products. The major contribution of this compound to smoothie formulations is due to the presence of strawberries. One serving of BF or FF (250 mL) provides about 483 μg of α-tocopherol. The recommended daily intake (RDI) of α-tocopherol for boys and girls aged 4–18 years is 11–15 mg per day and for men and women aged 19–50 years it is 12 mg per day [63]. Therefore, the intake of one serving of these formulations could cover around 4% of the RDI.

In the present study, the application of 450 MPa and 600 MPa did not alter α-tocopherol levels compared to the untreated sample. Table 2 shows that this compound was retained after HPP (450 MPa and 600 MPa) treatment in both formulations compared to the untreated samples (*p* ≤ 0.05). In agreement, α-tocopherol content was also retained in acai juice subjected to 450 and 600 MPa for 5 min at 20 °C [64]. In a study published by Barba et al. [65] investigating the impact of HPP application on a vegetable beverage (primarily tomato, green pepper, green celery) and orange juice with skimmed milk, the application of 200, 300, and 400 MPa for 9 min did not result in significant changes (*p* > 0.05) in the α-tocopherol content in the vegetable beverage. Conversely, a slight but significant decrease (*p* ≤ 0.05) was observed in orange juice with skimmed milk after the application of pressures higher than 200 MPa. Other studies showed that the stability of α-tocopherol when subjected to high-pressure processing is variable and no clear trend can be established, and this variability is often attributed to the plant matrix on which the processing is performed [65]. For example, significant decreases (*p* ≤ 0.05) were found when kale was subjected to 600 MPa for 10 min and for 40 min [31,66], where it was suggested that losses of the compound could be due to the effect of the pre-treatment of the kale (crushing or grinding) and the degree of homogenization of the samples prior to HPP application. On the other hand, in the study by Westphal et al. [53] on spinach and rosehip puree, significantly elevated concentrations (*p* ≤ 0.05) were found in spinach subjected to 200 MPa for 5 and for 10 min and to 400 MPa for 5 min and significant decreases (*p* ≤ 0.05) in rosehip puree subjected to 200 MPa for 5 min and to 400 MPa for 5 min. 

After 28 days of storage at 4 °C, significant differences (*p* ≤ 0.05) in the α-tocopherol contents were observed between HPP samples and untreated smoothies (Table 2). For smoothies treated at 450 MPa, BF showed a reduction of 49% and FF showed a reduction of 45%. No significant difference was determined by the comparison of BF and FF smoothies with regard to total α-tocopherol loss. Moreover, in smoothies treated at 600 MPa, it was observed that BF showed a significant reduction of 59% and FF showed a reduction of 60%. Again, this slight difference in percentage between BF and FF smoothies was not significant at the end of storage (*p* > 0.05). In BF, there was no significant difference (*p* > 0.05) in the percentage reductions for the different pressure conditions applied. However, in FF, it was observed that at 600 MPa, the percentage reduction was significantly higher (*p* ≤ 0.05) compared to 450 MPa. Few studies have been carried out to investigate the effects of storage on α-tocopherol content in plant matrices. In agreement, in kale subjected to 600 MPa for 10 min and stored for 2 months at 5 °C, a significant decrease of 90% in α-tocopherol was observed [66]. 

### 2.4. Bioaccessibility

For the addition of carrot fiber powder to the smoothies, no significant differences were found before and after HPP between the percentage reductions of each carotenoid contained in BF and FF, which suggests that the addition of this small amount (0.5%) of fiber from discarded carrots would not affect the bioaccessibility of each compound before and after the application of HPP. On the other hand, as mentioned above, dietary fiber plays an important role in the bioaccessibility of lipophilic compounds [38]. Table 3 shows that in our study, we found medium and low bioaccessible percentages, between 50 and 16%, of the individual carotenoids. The low bioaccessibilities found could be caused by the high natural fiber content of the formulations provided by the fruit components. The fiber present in the matrix is a food component that could affect the bioaccessibility of carotenoids, mainly by increasing the viscosity of the intestinal contents, trapping the bioactive compounds, and thus inhibiting the action of bile salts and lipases, preventing the carotenoids from being released from the food matrix and from being micellarized. Thus, the micellization and bioaccessibility of carotenoids was reduced due to the fiber content in the food matrix [67]. These findings explain the moderate and low bioaccessibility percentages of the carotenoid compounds identified in the undigested samples and the samples after in vitro digestion which exhibited higher bioaccessibility percentages.

Before processing, α-carotene showed a bioaccessibility of 50% in BF and 78% in FF. However, after the application of 450 MPa, the α-carotene content was reduced to 25% in BF and 19% in FF and, after the application of 600 MPa, it reduced to 34% in BF and 18% in FF. Notably, α-carotene continued to demonstrate the highest levels of bioaccessibility. This behavior with regard to α-carotene was also highlighted by Hacke et al. [68] in a study on fruit-based baby food, where a highly positive correlation was observed between the fiber content of the product and the bioaccessibility of α-carotene, which was attributed to the preprocessing or processing of the product (cutting, homogenization, etc.) that may have favorably modified the structure of the fibers, altering the solubility and interaction of α-carotene (which has a ring in the anterior plane (ε-ring)) with the fibers, and thus reducing its action on the bioaccessibility of this carotenoid. Another aspect to consider when analyzing the different bioaccessibilities of each compound is the structure of each carotenoid. The hydrophilicity of carotenoids provides a more efficient transfer to micelles, so xanthophylls showed a higher bioaccessibility than carotenes [38]. Consistent with this hypothesis, a study conducted with mango and papaya juices during simulated digestion positively correlated the relative efficiency of carotenoid micellization with their hydrophilicity, i.e., bioaccessibility of lutein > β-cryptoxanthin > β-carotene [39]. In our research, we uncovered unexpected outcomes wherein bioaccessibility declined in the following sequence: α-carotene, lutein, β-carotene, zeaxanthin and β-cryptoxanthin. This trend deviates from the initial hypothesis. A possible explanation could be that the experiments on which this hypothesis was developed considered individual fruit and vegetable purees, and did not consider more complex matrices formed from several individual foods. The mixing process could induce additional barriers or networks in addition to the natural barriers to carotenoid bioaccessibility. In fruit-based baby foods, the bioaccessibility of β-cryptoxanthin was significantly lower (*p* ≤ 0.05) than β-carotene, and this result was related to the composition of the product and the interaction between matrix components [68].

The impact of product processing on the bioaccessible percentage of each individual carotenoid can be seen in Table 3. Processing significantly influenced the individual bioaccessibility of each carotenoid, causing a significant decrease in their respective percentages.

After HPP treatment, the bioaccessibility of zeaxanthin appeared to be maintained in both formulations and both pressure conditions (*p* > 0.05). The bioaccessibility percentage of β-carotene in BF was also retained, with no changes after processing. The bioaccessibility of β-carotene was also retained in a kale-based juice (60% processed kale leaves in water and 40% apple juice) after processing at 500 MPa for 3 min [69].

On the other hand, a significant reduction (*p* ≤ 0.05) in the bioaccessibility of lutein was observed, with a decrease of approximately 50% after processing for BF and FF formulations compared to the content of the untreated sample. Regarding β-cryptoxanthin, BF showed a 48% reduction after the application of 450 MPa and a 31% reduction after the application of 600 MPa, while FF showed a 50% reduction after the application of 450 and 600 MPa. For α-carotene, BF showed a 50% reduction after the application of 450 MPa and a 32% reduction after the application of 600 MPa, while FF showed a 75% reduction after the application of both pressure conditions. Finally, β-carotene showed a 61% reduction after the application of 450 and 600 MPa. Cilla et al. [70] related this negative effect of HPP application on carotenoid bioaccessibility to some vegetables having firmer cell structures that require a higher processing pressure, with changes in the pulp microstructure after processing that can form a network of fibers that traps the carotenoid, making it less accessible to digestive enzymes and bile salts. In fruit beverages based on skim milk treated at 400 MPa for 5 min, Cilla et al. [71] found the same trend of a decrease in individual carotenoids. Thus, the bioaccessibility of zeaxanthin, lutein and β-cryptoxanthin underwent a significant decrease of 35% compared to their respective control beverages [71].

Regarding the impact of storage on the bioaccessibility of individual carotenoids, a predominantly positive trend was identified. As demonstrated in Table 3, the bioaccessibility of the carotenoid compounds, in most cases, either remained stable or exhibited an increase over the duration of storage. For lutein, despite the compound degradation during the storage period, its bioaccessibility increased during this period.

In addition, a statistical analysis (Table S1) indicated that no significant differences (*p* > 0.05) were found between untreated and treated smoothies at the end of storage for lutein, except in the FF treated at 450 MPa, where a significant decrease (*p* ≤ 0.05) of 7% in bioaccessibility was found. For zeaxanthin, despite the degradation observed during storage (Table 2), its bioaccessibility increased over this period (Table 3), and no significant differences (*p* > 0.05) were found between untreated and treated smoothies at the end of storage, except in the case of BF treated at 600 MPa (Table S1), where a significant decrease (*p* ≤ 0.05) of approximately 41% in its bioaccessibility was noted. In general, storage time had a positive effect on the bioaccessibility of β-cryptoxanthin (*p* > 0.05) (Table 3). Regarding α-carotene, in BF, it was observed that the application of HPP initially decreased the bioaccessibility of the compound on day 0, but then it increased during storage (Table 3). Thus, at day 28, no significant differences were found between untreated BF and these treated smoothies at the end of storage (Table S1). On the other hand, a significant decrease in bioaccessibility (*p* ≤ 0.05) was observed between the untreated FF and these smoothies treated at the end of storage—51% for 450 MPa and 43% for 600 MPa (Table S1). Finally, for β-carotene, a trend like that of α-carotene was observed in BF. Again, the application of HPP decreased the bioaccessibility of the compound at day 0, but then it increased during storage (Table 3). Thus, at day 28, no significant differences were found between untreated BF and these treated smoothies at the end of storage (Table S1). However, a significant loss of bioaccessibility (*p* ≤ 0.05) was observed between the untreated FF and treated FF smoothies at the end of storage—34% for 450 MPa and 24% for 600 MPa (Table S1). The preservation or increased bioaccessibility of β-carotene could be attributed to the effect that high-pressure processing has on the cell walls of the product, favoring the release of the compounds from the plant matrix and favoring the formation of micelles [38,65]. Further studies on the effect of storage on the bioaccessibility of individual carotenoids are needed.

After digestion, the bioaccessibility of α-tocopherol in untreated samples was 21% for BF and 23% for FF, with no significant differences (*p* > 0.05) between both formulations. As shown in Table 3, the application of HPP leads to small, not significant percentage variations (*p* > 0.05) in the bioaccessibility of this compound. In fruit beverages with milk and soy milk, the bioaccessibility of α-tocopherol was maintained after the application of 400 MPa for 5 min [71]. Table 3 demonstrates that during storage, a decline in bioaccessibility was observed for smoothies treated at 450 MPa, resulting in a mean loss of 42%, with no significant differences between the formulations. In contrast, smoothies treated at 600 MPa exhibited a significant increase in bioaccessibility at the end of storage, resulting in a mean increase of 67%, with no significant differences between the formulations. This significant increase could be because the higher pressure may have further modified the food matrix by modifying the location of tocopherol in the smoothies, changing their physicochemical states or altering the amounts of absorption effectors (fibers, proteins, etc.), thus making these lipophilic compounds more available for incorporation into micelles after gastrointestinal digestion and during storage [71].

## 3. Materials and Methods

### 3.1. Chemicals

All chemicals were of analytical grade. Solvents for use in HPLC, extraction procedures and to dissolve reference standards were obtained at HPLC-grade quality. All aqueous solutions were prepared by using HPLC-grade water (18 MΩ) from a Barnstead MicroPure UV system (Thermo Electron LED GmbH, Niederelbert, Germany). Carotenoid standards (97–99%) were purchased from CaroteNature (Münsingen, Switzerland). Pure tocopherols (>95%) were obtained from Calbiochem (Darmstadt, Germany). Pyrogallol (≥99%), magnesium carbonate basic (≥40% as MgO) and 2,6-di-tert-butyl-4-methyl-phenol (≥99%) were purchased from Sigma-Aldrich (Taufkirchen, Germany). Additionally, α-amylase (≥5 units/mg solid), pepsin (≥250 units/mg solid) and pancreatin (8 × USP) from porcine pancrease were obtained from Sigma-Aldrich. Porcine bile extract was purchased from Santa Cruz Biotechnology (Heidelberg, Germany). Peanut oil was obtained from a local grocery store. Sodium chloride (99.5%) was purchased from Fisher Scientific (Nidderau, Germany). Hydrochloric acid (32%), sodium bicarbonate (≥99.5%) and sodium sulfate (≥99%) were obtained from Carl Roth (Karlsruhe, Germany). 

### 3.2. Preparation of Smoothies

The base formulation (BF) of the smoothies was prepared by combining frozen strawberries (40% *w*/*w*), fresh orange juice without pulp (40% *w*/*w*), banana (10% *w*/*w*) and apple without peel (10% *w*/*w*), all purchased from a local supermarket (Jena, Germany). Fruits were selected by removing damaged fruits. Oranges, apples and bananas were washed separately with tap water for 1 min, disinfected via immersion in sodium hypochlorite 80 mg/L for 3 min (ratio of volume of disinfectant solution to weight of fruit: 5 L/kg). The juice was extracted from the oranges with a hand-operated fruit juicer and the pulp was removed from the juice with a manual strainer. The orange juice, frozen strawberries, peeled apples and bananas were placed together in a collecting container. All ingredients were processed using a fruit processor (SilverCrest SSMS 600 E6, produced for Lidl, Neckarsulm, Germany). The BF formulation was selected by establishing a fruit combination where one serving of the smoothie (200 g smoothie) would provide 100% of the recommended daily intake of vitamin C (≈90 mg ascorbic acid) [72]. The other formulation used was prepared by modifying BF with the addition of 0.5% carrot fiber powder to obtain a formulation with fiber (FF), reducing the percentage of the apple and banana content, as these are the fruits that contribute the least vitamin C to the smoothie. 

Carrot fiber powder was obtained by subjecting industrially discarded carrot bagasse to a solvent extraction, drying and milling process according to Patent AR099281B1 [73]. The composition of the powder obtained after drying this by-product was 73.6% *w*/*w* of total fiber (54.0% *w*/*w* of insoluble fiber and 19.6% *w*/*w* of soluble fiber), 7.1% *w*/*w* of protein, 7.0% *w*/*w* of ash, 0.2% *w*/*w* of fat and 11.4% *w*/*w* of moisture [21]. 

The initial soluble solids (SS) and pH were measured in the untreated samples (mean ± SD) (*n* = 3). Both formulations, BF and FF, had an initial SS content of 10.2 ± 0.1% and a pH of 3.4 ± 0.1. After HPP treatment and throughout storage, the SS and pH values were maintained without significant practical modifications.

The smoothies were manually bottled in 50 mL polyethylene terephthalate (PET) juice bottles with guarantee caps (Plasticflessen nl B.V., Zuidbroek, The Netherlands) for processing.

### 3.3. High-Pressure Processing (HPP) and Refrigerated Storage

Bottles (50 mL) containing the smoothie were treated via HPP in a Uhde 350-60 installation (Uhde High Pressure Technologies GmbH, Hagen, Germany), in a 350 L vessel equipped with a three-pump system, each with 2 intensifiers (model HPP D6090, Pmax = 600 MPa, Uhde High Pressure Technologies GmbH, Hagen, Germany). A batch of 50 bottles of BF and 50 bottles of FF were treated at 450 MPa for 3 min. Another batch was performed with the same amount and type of product, but at 600 MPa for 3 min. The time taken to obtain the working pressure was 120 s and 170 s for 450 and 600 MPa, respectively, and the decompression time was approximately 15 s. The initial water temperature was adjusted to 11.00 ± 0.20 °C in the high-pressure vessel. Each pressure condition was performed once, since a higher number of replicates would have incurred additional operational costs and difficulties in terms of the availability of industrial equipment. The HPP-treated samples were stored at 4 °C for 28 days. 

The microbiological assays were performed according to DIN EN ISO 4833-2 (Microbiology of the food chain—Horizontal method for the enumeration of microorganisms—Part 2: Colony count at 30 °C by the surface plating technique) [74] by a private laboratory accredited by the Deutsche Akkreditierungsstelle GmbH DAkkS according to DIN EN ISO/IEC 17025:2018. Microbiological safety and quality were maintained for 60 days with the absence of pathogens (*Escherichia coli* and *Listeria monocytogenes*) and a spoilage microorganism count under 100 CFU/g (aerobic mesophilic, yeasts, molds and lactic acid bacteria), complying with the requirements of Regulation No 2073 (2005) of the European Commission and the Food and Drug Administration (FDA) (FDA, 2004).

After each storage period, a triplicate of each sample was freeze-dried for 48 h. The freeze-dried samples were used to prepare smoothie extracts.

### 3.4. Determination of Carotenoids and Vitamin E Content

#### 3.4.1. Extraction Method

The extraction was performed according to Böhm (2011) [41] with slight modifications. Dried smoothie samples (5 g) were weighed into 50 mL conical test tubes. Then, 200 mg of magnesium carbonate, 200 mg of sodium sulphate and internal standard volumes (250 μL of lycopene and 50 μL of δ-tocotrienol) were added. Then, 30 mL of a methanol-tetrahydrofuran (MeOH/THF) mixture (50:50 = *v*/*v*), including 0.1 wt.% butylated hydroxytoluene (BHT), was used as an extraction solvent and vortexed for 1 min. All the samples were sonicated three times in an ice bath under reduced daylight conditions and centrifuged at 3500× *g* for 5 min at 4 °C for phase separation between repeat extractions. The combined upper phases were evaporated under reduced pressure using a rotary evaporator at 30 °C. The residue was dissolved in MeOH/MtBE (70:30 = *v*/*v*) and used after centrifugation at 18,500× *g* for 5 min for subsequent HPLC analysis.

#### 3.4.2. Saponification

Due to the presence of esters in the smoothie extracts, saponification is required to hydrolyze them before HPLC analysis of carotenoids. Saponification in BF and FF extracts was conducted according to Böhm [41] with some modifications. Briefly, 2 mL of 10% methanolic KOH solution was added to 2 mL of extract and allowed to stand for 90 min at room temperature under subdued light. Then, 0.5 mL of water and 2 mL of petroleum ether were added, mixed for 1 min in vortex and centrifuged at 3500× *g* for 1 min. The upper layer containing the carotenoids was transferred to another test tube. The lower hydrophilic layer was extracted four times with 2 mL of petroleum ether each time until the organic layer was colorless. Subsequently, the combined organic phases were washed several times with water to remove KOH. The petroleum ether solution was dried under vacuum at 30 °C using a rotary evaporator. The residue was dissolved in MeOH/THF solution (50:50 = *v*/*v*), including 0.1 wt.% BHT, until the solution reached a volume of 2 mL. The solution was centrifuged at 18,500× *g* for 5 min for HPLC analysis.

#### 3.4.3. HPLC-DAD

The saponified extracts were analyzed by using a VWR Hitachi Chromaster (5000 series) reversed-phase HPLC system (Develosil C30, 250 × 4.6 mm, 5 µm, Phenomenex, Aschaffenburg, Germany) at a column temperature of 13 °C and an injection volume of 50 µL. Both an eluent gradient and a flow gradient were applied. At 0 min, the eluent gradient started at 9% of solvent A (MeOH) and 91% of solvent B (MtBE), at a flow rate of 0.43 mL/min. Solvent A was then increased to 50% over 23.5 min at constant flow rates. Afterwards, solvent A was increased to 70% until 38 min, with an increasing flow rate of 0.6 mL/min, which was held until 50 min. Subsequently, solvent A was reduced to 9% at constant flow rates. At minute 58, the flow rate was increased to 1.00 mL/min, and after an equilibrium holding time of 7 min for equilibration, a diode array detector was used for identification at 450 nm. Carotenoid contents were quantified by 6-point calibration curves (r^2^ > 0.999) of external standards, using lycopene as an internal standard (recovery). The identifications were performed via the comparison of retention times and DAD absorbance spectra as well as mass spectra.

The limit of detection (LOD) and limit of quantification (LOQ) of each analyte were based on signal-to-noise ratios of S/N = 3:1 and S/N = 10:1, respectively, and were determined using the baseline noise signals in the chromatograms of 5 solvent injections.

#### 3.4.4. HPLC-MS

LC-MS analysis was performed by using an API 2000 MS/MS system (AB Sciex, Darmstadt, Germany). A Shimadzu HPLC system (LC-20 series, Shimadzu, Duisburg, Germany) was used. Separation was achieved on a reversed-phase column (YMC C30, 250 × 4.6 mm, 5 µm, YMC Europe, Dinslaken, Germany), applying a gradient elution with MeOH/water (80:20, *v*/*v*; A) and MtBE/MeOH/water (78:20:2, *v*/*v*/*v*; B) at 30 °C. The Pumping flow mode was kept isocratic at 1.3 mL/min. The gradient elution started with an increase in solvent B to 30% for 5 min, and then increased to 60% until 35 min. Finally, solvent B was set to 100% until 42 min, which was maintained for 1 min afterwards. The re-equilibration time was set to 7 min at 100% of solvent A. MS measurements were performed in positive Q1 scanning mode, comparing external standards of carotenoids with compounds from smoothie extract. The MS parameters were as follows: nebulizer gas: nitrogen; nebulizer current: 1.5 µA; vaporizer temperature: 400 °C; declustering potential (DP): 35.0 V; fokussion potential (FP): 400.0 V; entrance potential (EP): 7.0 V. Analyst^®^ (Version 1.5.2, AB Sciex, Darmstadt, Germany) was applied for data evaluation.

#### 3.4.5. Identification and Quantification of Vitamin E

Vitamin E analysis of extracts (without saponification) was performed via normal phase chromatography using a Jasco LC-900 series HPLC (JASCO Deutschland GmbH, Pfungstadt, Germany) system and fluorescence detection (NP-HPLC-FLD). Thus, 400 μL of the extract previously redissolved in MeOH/MtBE (70:30 = *v*/*v*) was subjected to solvent exchange under nitrogen at 30 °C into a mixture of 400 μL of n-hexane/MtBE (98:2 = *v*/*m*), which was also used for isocratic elution. Then, 50 μL of the BF and FF extracts were injected onto a Eurospher Diol column (250 × 4.6 mm, 5 μm, Knauer, Berlin, Germany) with a set flow rate of 1.5 mL/min at 25 °C for 40 min. α-Tocopherol was identified via the comparison of the retention times with the corresponding external standard. Quantification was achieved with a 6-point calibration curve (r^2^ > 0.999) and considering the recovery rates of the internal standard (δ-tocotrienol). Linearity was provided over the entire 6-point calibration curve. Excitation and emission wavelengths were set at 292 nm and 330 nm. Jasco ChromNav (Version 1.18.07, Build 3) was applied for data evaluation.

### 3.5. In Vitro Digestion Model

#### 3.5.1. Experimental Procedure

A static in vitro digestion model was proposed for BF and FF samples, adapted from Reboul et al. [37], Werner and Böhm [42] and Minekus et al. [43], considering the type of food matrix used in this case. 

**Figure 2:**
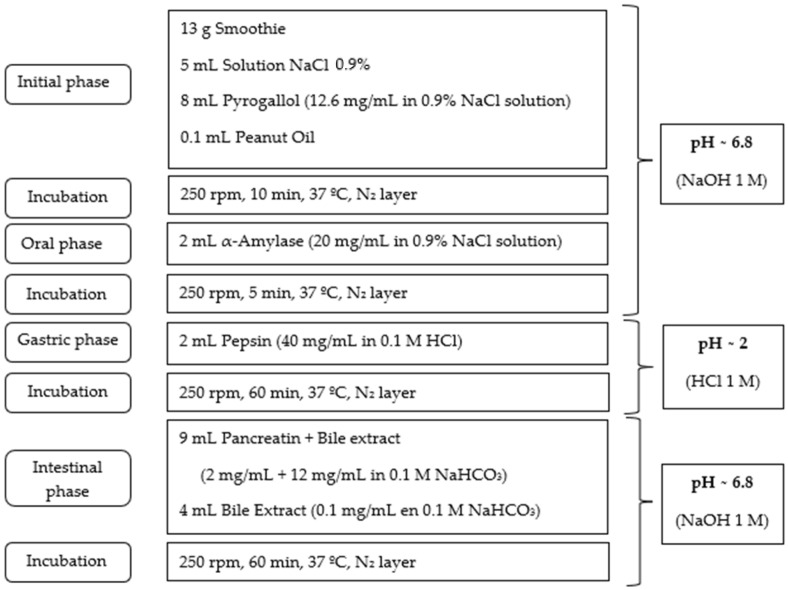
Experimental procedure of in vitro digestion.

About 13 g of fresh BF and FF were weighed into 100 mL stoppered Erlenmeyer flasks that served as containers for digestion. The initial phase was placed in the container to properly mix the fresh smoothie, NaCl solution, peanut oil to promote carotenoid micellarization and pyrogallol to prevent oxidation of the carotenoid compounds. An orbital shaker-incubator Grant-bio ES-20 (Grant instruments, Rayston, UK) was used at 250 rpm and 37 °C under reduced daylight conditions. The mixtures of each phase were covered with nitrogen for incubation. The adjustment of pH values was achieved via the addition of predefined volumes of 1 M NaOH and 1 M HCl solutions, as shown in Figure 2. 

#### 3.5.2. Isolation of the Micellar Fraction

Digested samples were centrifuged at 3382× *g* at 4 °C for 20 min to separate the fraction of analytes released from the food matrix. The supernatant then was divided into aliquots which were transferred to 2 mL test tubes and placed in a centrifuge at 18,500× *g* for 5 min. Cellulose filters with a 0.45 µm pore size were used to filter the supernatant. The filtered supernatant was stored in a freezer for subsequent freeze-drying.

#### 3.5.3. Extraction of Carotenoids and Vitamin E

The extraction of carotenoids and vitamin E was carried out in line with the procedures performed by Werner and Böhm [42] with minor modifications. Around 0.6 g of freeze-dried supernatant was weighed into test tubes and 2 mL of MeOH/MtBE (70:30 = *v*/*v*) and volumes of internal standards (40 μL of lycopene and 20 μL of δ-tocotrienol) were added. After 30 s of vortexing, the samples were sonicated for 5 min. Subsequently, 1.5 mL aliquots were centrifuged at 18,500× *g* for 5 min at room temperature for subsequent HPLC-DAD analysis of carotenoids. Finally, an additional 400 µL aliquot was dried under a stream of nitrogen at 30 °C and dissolved in 400 µL of n-hexane/MtBE (98:2 = *v*/*m*), followed by centrifugation at 18,500× *g* for 5 min prior to HPLC analysis of vitamin E.

#### 3.5.4. Calculations

Bioaccessibility was calculated as the percentage of the content of carotenoids determined in the micellar aqueous fraction of the digesta after centrifugation and filtration in relation to the respective initial content in the undigested raw samples. The calculations were based on the following formula:

(1) $$\mathrm{Bioaccessibility}\;\%=\frac{C_{\mathrm{Micella}}}{C_{\mathrm{Raw}}}\times 100$$

where C_Micella_ and C_Raw_ are the concentrations of carotenoids in the micellar fraction and in the undigested raw sample, respectively.

### 3.6. Statistical Analysis

STATGRAPHICS Centurion XV (StatPoint Technologies Inc., Warrenton, VA, USA) was used for analyzing data through ANOVA and significant differences between means were determined by means of Tukey’s test at a 5% significance level (*p* ≤ 0.05).

## 4. Conclusions

This study demonstrates that both pressure processing conditions used (450 MPa/3 min and 600 MPa/3 min) can be advantageous for smoothies to retain or even increase the contents of natural bioactive compounds, probably due to the improvement of their extractability. The application of HPP in smoothies increased the contents of pro-vitamin A carotenoid compounds (β-carotene, α-carotene and β-cryptoxanthin) and retained the contents of lutein, zeaxanthin and α-tocopherol when compared to untreated samples. These results imply the economic potential of using a gentler HPP treatment (450 MPa) than that used commercially (600 MPa) to reduce production costs while ensuring the health-promoting potential of the product. 

Although HPP application resulted in decreased bioaccessibility of carotenoids and vitamin E in BF and FF smoothies compared to untreated samples at day 0, the bioaccessibility of some compounds increased during storage. Specifically, the bioaccessibility of lutein and α-carotene in BF and FF at 450 MPa and 600 MPa increased during refrigerated storage. Moreover, the bioaccessibility of most lipophilic compounds in HPP-treated BF and FF after 28 days of refrigerated storage was similar to that of untreated smoothies. 

Although the effect of the addition of carrot fiber powder on the bioaccessibility of lipophilic compounds in HPP-treated smoothies needs to be further investigated, the incorporation of this food additive from industrial residues promotes circular economy practices and increases the health potential of this type of beverage.

## Figures and Tables

**Table 1:** Identified compounds in smoothies with experimental parameters derived from external standard measurements.

N°	Compound Name	t_R_ (min)	λ_max 1_ (nm)	λ_max 2_ (nm)	λ_max 3_ (nm)	*m*/*z* [M + H]^+^
1	(*all-E*)-Antheraxanthin	17.46	422	445	473	585.2
2	(*all-E*)-Lutein	19.21	421	445	473	551.1
3	(*all-E*)-Zeaxanthin	20.41	428	451	478	569.2
4	(*all-E*)-β-Cryptoxanthin	29.24	-	452	478	553.2
5	(*all-E*)-α-Carotene	35.46	421	447	475	537.3
6	(*all-E*)-β-Carotene	36.74	428	452	479	537.3

t_R_: retention time.

**Table 2:** Carotenoids and α-tocopherol contents (μg/100 g) of untreated and high-pressure processed smoothie and its evolution during storage at 4 °C (mean ± SD) (*n* = 3).

**Treatment**	**Storage** **(Days)**	**Lutein** **(μg/100 g)**		**Zeaxanthin** **(μg/100 g)**		**β-Cryptoxanthin** **(μg/100 g)**	
		**BF**	**FF**	**BF**	**FF**	**BF**	**FF**
Untreated	0	12.6 ± 1.1 A	14.4 ± 1.6 A	6.0 ± 0.5 A	7.0 ± 1.2 A	14.5 ± 0.9 A	10.7 ± 0.6 A
450 MPa	0	16.8 ± 1.4 Ab	11.9 ± 1.5 Ab	8.0 ± 1.3 Ab	5.9 ± 1.2 Ab	19.3 ± 0.7 Bc	12.0 ± 0.3 Bb
	14	12.0 ± 0.5 a	13.2 ± 0.5 b	6.1 ± 0.5 a	5.6 ± 0.36 b	16.4 ± 0.1 b	12.7 ± 0.2 c
	28	10.4 ± 0.8 a	9.6 ± 0.7 a	5.3 ± 0.2 a	4.6 ± 0.5 a	12.3 ± 0.7 a	9.1 ± 0.1 a
600 MPa	0	19.7 ± 4.5 Ab	15.8 ± 1.7 Ab	9.6 ± 2.8 Ab	6.7 ± 1.4 Ab	20.3 ± 2.6 Bb	13.1 ± 0.8 Bb
	14	15.0 ± 0.9 ab	14.6 ± 0.7 b	6.8 ± 0.4 ab	6.8 ± 0.3 b	17.6 ± 0.7 b	17.6 ± 0.5 c
	28	8.8 ± 2.9 a	8.7 ± 2.4 a	4.2 ± 1.0 a	4.1 ± 1.3 a	10.2 ± 2.9 a	8.3 ± 2.6 a
**Treatment**	**Storage** **(Days)**	**α-Carotene** **(μg/100 g)**		**β-Carotene** **(μg/100 g)**		**α-Tocopherol** **(μg/100 g)**	
		**BF**	**FF**	**BF**	**FF**	**BF**	**FF**
Untreated	0	3.9 ± 0.6 A	3.0 ± 0.3 A	6.9 ± 0.7 A	6.8 ± 0.5 A	187.9 ± 19.2 A	198.2 ± 8.8 A
450 MPa	0	5.5 ± 0.5 Bb	4.0 ± 0.6 Ba	9.2 ± 0.3 Bc	7.4 ± 0.1 Ab	183.8 ± 7.7 Ab	181.6 ± 7.9 Ac
	14	4.3 ± 0.5 a	3.8 ± 0.3 a	6.8 ± 0.1 b	7.4 ± 0.3 b	103.9 ± 8.6 a	146.7 ± 7.5 b
	28	3.0 ± 0.3 a	3.0 ± 0.3 a	5.3 ± 0.5 a	5.4 ± 0.2 a	97.0 ± 19.0 a	109.8 ± 2.12 a
600 MPa	0	5.7 ± 1.0 Bb	4.5 ± 0.7 Bb	8.9 ± 1.3 Bb	8.2 ± 0.5 Bb	192.0 ± 3.7 Ac	186.6 ± 6.5 Ab
	14	4.7 ± 0.5 b	4.6 ± 0.5 b	7.4 ± 0.6 b	7.3 ± 0.5 b	136.3 ± 4.8 b	175.0 ± 6.2 b
	28	2.9 ± 0.4 a	2.6 ± 0.4 a	4.9 ± 1.0 a	5.0 ± 0.8 a	77.0 ± 3.9 a	78.7 ± 12.3 a

BF: smoothie base formulation, FF: smoothie with dietary fiber. Different capital letters in the same column indicate significant differences (*p* ≤ 0.05) between the untreated and treated sample at different pressures on day 0. Different lowercase letters in the same column indicate significant differences (*p* ≤ 0.05) during storage for each HPP treatment.

**Table 3:** Bioaccessibility of carotenoids and α-tocopherol (%) in untreated and high-pressure processed smoothies and its evolution during storage at 4 °C (mean ± SD) (*n* = 3).

Bioaccessibility (%)							
**Treatment**	**Storage** **(Days)**	**Lutein**		**Zeaxanthin**		**β-Cryptoxanthin**	
		**BF**	**FF**	**BF**	**FF**	**BF**	**FF**
Untreated	0	30.4 ± 7.0 B	31.7 ± 2.8 B	19.3 ± 3.2 A	22.5 ± 2.3 B	15.7 ± 3.0 B	22.0 ± 4.2 B
450 MPa	0	15.2 ± 3.1 Aa	19.8 ± 2.8 Ab	12.7 ± 2.1 Aa	14.5 ± 1.6 Aa	9.5 ± 0.9 Aa	10.3 ± 3.7 Aa
	14	28.7 ± 2.2 b	26.8 ± 1.5 ab	18.5 ± 2.0 b	21.6 ± 5.7 a	14.9 ± 0.3 b	15.0 ± 1.2 a
	28	29.1 ± 1.8 b	24.9 ± 2.0 a	21.4 ± 1.0 b	18.6 ± 3.9 a	15.4 ± 0.8 b	14.9 ± 1.6 a
600 MPa	0	18.0 ± 5.9 Aa	20.7 ± 4.6 Aa	15.1 ± 4.1 Aa	16.0 ± 3.1 Aa	11.1 ± 2.5 ABa	11.2 ± 5.1 Aa
	14	25.7 ± 1.7 ab	23.1 ± 2.5 a	17.0 ± 2.9 a	16.0 ± 4.3 a	13.7 ± 0.6 a	9.4 ± 1.6 a
	28	32.6 ± 3.0 b	32.4 ± 1.3 b	11.5 ± 2.5 a	20.1 ± 1.1 a	25.1 ± 3.5 b	18.4 ± 0.8 b
**Treatment**	**Storage** **(Days)**	**α-Carotene**		**β-Carotene**		**α-Tocopherol**	
		**BF**	**FF**	**BF**	**FF**	**BF**	**FF**
Untreated	0	50.2 ± 2.4 C	77.6 ± 4.8 B	26.9 ± 6.6 A	38.5 ± 2.3 B	21.3 ± 2.2 A	23.3 ± 5.0 A
450 MPa	0	25.3 ± 1.5 Aa	19.3 ± 7.5 Aa	16.1 ± 1.6 Aa	14.9 ± 2.6 Aa	21.3 ± 3.1 Ab	24.5 ± 5.5 Ab
	14	46.0 ± 5.5 b	42.6 ± 4.1 b	29.2 ± 0.4 b	25.6 ± 2.2 b	29.1 ± 4.4 c	18.3 ± 2.7 ab
	28	47.4 ± 3.1 b	37.8 ± 5.0 b	28.9 ± 2.4 b	24.9 ± 1.2 b	12.5 ± 2.3 a	13.7 ± 1.7 a
600 MPa	0	33.8 ± 4.2 Ba	17.6 ± 8.2 Aa	22.2 ± 4.1 Aa	14.8 ± 4.0 Aa	21.0 ± 2.7 Aa	18.4 ± 2.6 Aa
	14	42.0 ± 4.5 a	18.5 ± 1.8 a	26.0 ± 2.0 a	18.5 ± 3.7 a	25.6 ± 2.9 a	15.1 ± 1.4 a
	28	61.1 ± 9.7 b	44.0 ± 8.1 b	36.1 ± 8.9 a	28.5 ± 3.7 b	37.2 ± 2.8 b	37.1 ± 6.3 b

BF: smoothie base formulation, FF: smoothie with dietary fiber. Different capital letters in the same column indicate significant differences (*p* ≤ 0.05) between the untreated and treated samples at different pressures on day 0. Different lowercase letters in the same column indicate significant differences (*p* ≤ 0.05) during storage for each HPP treatment.

## Data Availability

The data presented in this study are included in the article.

## Supplementary Materials

The following supporting information can be downloaded at: https://www.mdpi.com/article/10.3390/molecules29061259/s1, Table S1: Bioaccessibility of carotenoids and α-tocopherol (%) of untreated and high-pressure-processed smoothies after 28 days of storage at 4 °C (mean ± SD) (*n* = 3).
