# Supplementary material for: High-Pressure Processing of Fruit Smoothies Enriched with Dietary Fiber from Carrot Discards: Effects on the Contents and Bioaccessibilities of Carotenoids and Vitamin E
Source: Molecules. 2024 Mar 12;29(6):1259. doi: 10.3390/molecules29061259 (PMC10974493; doi:10.3390/molecules29061259)
Supplement: Supplementary file 1 [file molecules-29-01259-s001.zip › molecules-2841189-supplementary.pdf]

Supplementary Material

High-Pressure Processing of fruit smoothies enriched with dietary fiber from carrot discards: effects on the contents and bio-accessibilities of carotenoids and vitamin E.

Authors: Melisa Donda Zbinden [1,2], Mario Schmidt [3], Charito Ivana Vignatti [1,2], Maria Élida Pirovani [1] and Volker Böhm [3,*]

Affiliations:

[1] Instituto de Tecnología de Alimentos, Universidad Nacional del Litoral, 3000 Santa Fe, Argentina

[2] Consejo Nacional de Investigaciones Científicas y Técnicas (CONICET), 3000 Santa Fe, Argentina

[3] Institute of Nutritional Sciences, Friedrich Schiller University Jena, 07743 Jena, Germany

Corresponding author:

[*] Institute of Nutritional Sciences, Friedrich Schiller University Jena, Dornburger Straße 25-29, 07743 Jena, Germany. E-Mail address: volker.boehm@uni-jena.de, Tel.: +49 3641 949633; fax: +49 3641 949702.

Table S1. Bioaccessibility of carotenoids and α-tocopherol (%) of untreated and high-pressure processed smoothies after 28 days during storage at 4 ºC (mean ± SD) (n=3).

Treatment	Storage (days)	Lutein		Zeaxanthin		β-cryptoxanthin	
		**Bioaccessibility (%)**					
		BF	FF	BF	FF	BF	FF
Untreated	0	30.4 ± 7.0Aa	31.7 ± 2.8Ba	19.3 ± 3.2Ab	22.5 ± 2.3Aa	15.7 ± 3.0Aa	22.0 ± 4.2Aa
450	28	29.1 ± 1.8A	24.9 ± 2.0A	21.4 ± 1.0A	18.6 ± 3.9A	15.4 ± 0.8A	14.9 ± 1.6A
600	28	32.6 ± 3.0a	32.4 ± 1.3a	11.5 ± 2.5a	20.1 ± 1.1a	25.1 ± 3.5b	18.4 ± 0.8a

Table S1. *Cont.*

Treatment	Storage (days)	α-carotene		β-carotene		α-tocopherol	
		BF	FF	BF	FF	BF	FF
Untreated	0	50.2 ± 2.4Aa	77.6 ± 4.8Bb	26.9 ± 6.6Aa	38.5 ± 2.3Bb	21.3 ± 2.2Ba	23.3 ± 5.0Ba
450	28	47.4 ± 3.1A	37.8 ± 5.0A	28.9 ± 2.4A	24.9 ± 1.2A	12.5 ± 2.3A	13.7 ± 1.7A
600	28	61.1 ± 9.7a	44.0 ± 8.1a	36.1 ± 8.9a	28.5 ± 3.7a	37.2 ± 2.8b	37.1 ± 6.3b

BF: Smoothie base formulation, FF: smoothie whit dietary fiber. Different capital letters in the same column indicate significant differences ($p \leq 0.05$) between the untreated and treated sample at 450 MPa after 28 days of refrigerated storage. Different lowercase letters in the same column indicate significant differences ($p \leq 0.05$) between the untreated and treated sample at 600 MPa after 28 days of refrigerated storage.
